# Genomic analysis of *Chlamydia psittaci* from a multistate zoonotic outbreak in two chicken processing plants

**DOI:** 10.7150/jgen.86558

**Published:** 2023-08-19

**Authors:** Bernard J. Wolff, Jessica L. Waller, Alvaro J. Benitez, Anna Gaines, Andrew B. Conley, Lavanya Rishishwar, Aroon T. Chande, Shatavia S. Morrison, I. King Jordan, Maureen H. Diaz, Jonas M. Winchell

**Affiliations:** 1 Division of Bacterial Diseases, Centers for Disease Control and Prevention, Atlanta, GA, USA; 2 Applied Bioinformatics Laboratory, Atlanta, GA, USA; 3 School of Biological Sciences, Georgia Institute of Technology, Atlanta, GA, USA

## Abstract

Four *Chlamydia psittaci* isolates were recovered from clinical specimens from ill workers during a multistate outbreak at two chicken processing plants. Whole genome sequencing analyses revealed high similarity to *C. psittaci* genotype D. The isolates differed from each other by only two single nucleotide polymorphisms, indicating a common source.

*Chlamydia psittaci* is an obligate intracellular bacterium with a unique biphasic life cycle that alternates between an infectious non-replicating elementary body (EB) form and a metabolically active replicating reticulate body (RB) form 1. This species is a member of the *Chlamydiace* family, which dates back to 50 to 250 million years ago, and has been shown to have a broad range of host species 2. While *C. psittaci* has traditionally been documented in psittacine birds, it has also been found in cattle, sheep, swine, goats, cats, horses, and at least 465 bird species spanning 30 different orders 2-4. It is the etiologic agent of psittacosis, a respiratory zoonotic infection in humans that can vary in severity and have multi-organ involvement, resulting in significant morbidity and mortality 5. While* C. psittaci* has been documented in turkeys for decades, evidence of its presence in chickens has only been recently documented 6, 7. There are ten well described *C. psittaci* genotypes designated A-G, E/B, M56 and W/C, which differ in virulence potential and host species tropism 2, 8. Genotype D is most commonly associated with poultry outbreaks and is extensively excreted relative to other genotypes 2, 6, 8, 9. While there are many publicly available genome sequences of *C. psittaci*, there are no whole genome sequences available for* C. psittaci* isolates originating from chickens. We performed whole genome sequencing analysis on isolates obtained from clinical specimens collected from ill workers during an outbreak at two chicken processing plants in 2018.

From August 31 to September 28, 2018, 50 chicken processing plant workers in Virginia and 30 in Georgia became ill with a respiratory illness, 29 of whom required hospitalization 10. Sputum, bronchoalveolar lavage washings (BAL), nasopharyngeal (NP) and oropharyngeal (OP) swabs, and stool specimens from plant workers meeting the case definition were submitted to the Centers for Disease Control and Prevention (CDC) and tested by real-time polymerase chain reaction (PCR) 10, 11. Thirteen patients had *C. psittaci* detected in one or more clinical specimens 11. Sequencing of the outer membrane protein A (*ompA*) gene was attempted on specimens with Ct values less than 30 (n=7) as previously described 11, 12. All specimens matched the *ompA* gene of genotype D with 100% pairwise identity. Culture was attempted on lower respiratory tract specimens (n=8) that were PCR positive, owing to the relatively higher *C. psittaci* concentrations previously found in this specimen type after inoculation of Vero cell monolayers 13. Isolates were obtained from four of eight specimens, with recovery rate being affected by bacterial and/or fungal contamination, lower pathogen load, and possible low viability due to transport and cell culture delays. Growth of *C. psittaci* was confirmed by observing cytopathogenic effects through microscopy and concomitant real-time PCR testing of cell culture supernatants 11. *Chlamydia* EBs were purified by renografin gradient centrifugation as previously described 14. Nucleic acid was extracted from the purified EBs using the MasterPure Complete DNA and RNA Purification Kit (Epicentre, Madison, WI) according to the manufacturer's instructions. Libraries were prepared using the Illumina Nextera XT library preparation kit per manufacturer's instructions and sequenced on a MiSeq System (Illumina, San Diego, CA) with version 2 500 cycle kits using 2x250 paired-end sequencing. Long read sequencing was performed using either Pacific Biosciences (PacBio) RS II (Menlo Park, CA) or Oxford Nanopore MinION (OX4 4DQ, UK) using Oxford Nanopore Rapid PCR Barcoding Kit (SQK-RPB004). Libraries were prepared using the PacBio SMRTbell Template Prep Kit 1.0 or the Oxford Nanopore Rapid PCR Sequencing Kit per the manufacturer's instructions. Long read sequencing was not performed for one isolate, VA1, due to contamination; however, Illumina MiSeq sequence data yielded a sufficiently high-quality genome for this isolate. PacBio reads for GA1, VA2 and VA3 were assembled using HGAP version 3, Oxford Nanopore MinION reads for GA1 were assembled using Flye 15, 16, Medaka 17, and Pilon 18, and Illumina reads for VA1 were assembled using SPAdes version 3.11.1 19. PacBio and MinION assemblies yielded finished chromosomal assemblies, while assembly of Illumina reads resulted in a nearly complete genome with 3 contigs (>1,000bp). No major differences were observed in the finished PacBio and Nanopore assemblies for the GA1 isolate. For whole genome comparisons, we used the PacBio assemblies for GA1, VA2, and VA3 and the draft Illumina assembly for VA1 (Table 1).

Each of the assembled genomes was compared to reference *C. psittaci* assemblies from NCBI using all-against-all pairwise average nucleotide identity (ANI) values, calculated by using the MUMmer program 20. All the isolates form a cluster with the reference genome NJ1, a *C. psittaci* genotype D isolate (NC_018626.1; Figure 1A). All the isolates were observed to be >99% similar to NJ1. ANI values were converted to distance measures (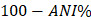) and used to construct a phylogenetic tree using the Neighbor-Joining algorithm 21 in the program Mega X 22. The phylogenetic comparison also supported clustering of the isolates with the NJ1 reference genome (Figure 1B). NJ1 was subsequently used throughout the study as the reference genome when performing additional analyses on these newly assembled genomes.

Whole genome alignment using Mauve version 20150226 build 10 23 demonstrated no major rearrangements between the assemblies and NJ1 (Supplementary Figure 1). Comparison of finished assemblies to the reference genome indicated a 361 bp insertion within the membrane protein gene (WP_014946495.1). This insertion was also detected in the genome of an additional reference isolate WC, indicating a deletion in NJ1 not shared by the isolates analyzed in this report. For variant analysis, Illumina MiSeq reads were mapped against the *C. psittaci* NJ1 genome using the program BWA 24 followed by variant calling with SAMtools 25, 26. The four outbreak isolates are nearly identical to each other, differing by at most two SNPs and are also highly similar to the NJ1 reference with only 61 SNPs and 2 insertions identified (Table 2).

Supplementary Table 1 lists all the observed nucleotide substitutions, positions, variant qualities, affected genes, and variant effects in these isolates. Variants were categorized by potential impact based on the nature of the mutation 27. One insertion and one variant were considered high impact because they resulted in a frameshift mutation and the loss of a stop codon. However, these mutations occurred in a hypothetical protein and a domain of unknown function, so the potential disruption of these mutations remains unclear. Thirty variants were classified as moderate variants causing missense mutations within genes. There were ten variants and one insertion that were intergenic modifying variants resulting in upstream gene variants in regulatory regions upstream of genes. Lastly, twenty variants resulted in a synonymous variant and thus were classified as low impact.

The isolates in this study were remarkably conserved relative to the genotype D reference strain NJ1, which was initially isolated from a turkey in 1954 2. Despite nearly 65 years between the original isolation and multiple passages of the original reference strain, the genomes of the outbreak isolates gained the equivalent of one variant per year since the original isolation of the reference genotype strain. However, due to the lack of representative genomes it cannot be determined how or when these variants were accumulated. In contrast to other intracellular bacterial species that demonstrate ongoing genome reduction and frequent horizontal gene transfer 19, our findings are consistent with the reported stability of Chlamydial genomes over time despite the ability to frequently jump host species 2. *C. psittaci* has been well documented in turkeys with genotype D being the most prevalent. Chickens have only recently been studied for the presence of *C. psittaci* in Europe, Australia and Asia 3, 6, 28-31, but to date no studies of its prevalence in chickens in the United States have been published. It is possible one or more of the variants identified in the outbreak isolates allowed genotype D to more easily infect chickens. This phenomena has been documented previously in other bacterial species; Viana *et al.* determined that one variant within the *dltB* gene responsible for lipoteichoic acid biosynthesis, an upstream precursor to cell wall biosynthesis, was solely responsible for the *Staphylococcus aureus* human-to-rabbit host jump over 40 years ago 32. Mutations impacting membrane proteins are of particular interest due to their interaction with host cell receptors, but other genes may also contribute to host specificity. Of the variants identified in this study that were of moderate impact or modifiers in intergenic DNA, two impact a perforin family protein and one is located in an outer membrane protein. All three variants could potentially impact host preference. Further research will be needed to investigate the infectivity of these isolates in chickens relative to other genotype D isolates.

Traditionally, it was conjectured that chickens are less susceptible to *C. psittaci* compared to turkeys, although recent outbreaks have brought this into question 6, 7. Differences in turkey and chicken processing operations were thought to be a contributing reason behind the lower observed rates of *C. psittaci* infection in chickens. Turkeys are typically raised for 15 to 17 weeks before they are slaughtered compared to 6 weeks for a typical chicken 3. The extended time in turkeys could allow *C. psittaci* to spread to a larger portion of the population and have more time to reach higher bacterial loads in the birds before slaughter. However, up until 2010 the only *C. psittaci* genotypes detected in chickens were the less virulent genotypes B, C, and E/B 7, 29, 33. Genotype D has been shown to be highly virulent and excreted extensively compared to other genotypes and has only recently been documented in chickens 3, 6. These characteristics may allow genotype D to more rapidly infect flocks in the close quarters of chicken houses. This genotype was identified early in the outbreak using sanger sequencing of the *ompA* gene following nucleic acid extraction from clinical specimens. From the initial *ompA* sequencing and knowing both plants were receiving chickens from the same farms, we were able to hypothesize that the outbreaks in both Georgia and Virginia were likely from the same source, and that it was a historically highly virulent genotype that required aggressive treatment and management to mitigate the outbreak. Additional* ompA* sequencing with next generation and third generation sequencing chemistries provided additional clues and insights into how *C. psittaci* may be evolving to more efficiently infect hosts and/or increase virulence and should be considered when investigating uncommon outbreaks.

## Supplementary Material

Supplementary figure and table.

## Figures and Tables

**Figure 1:**
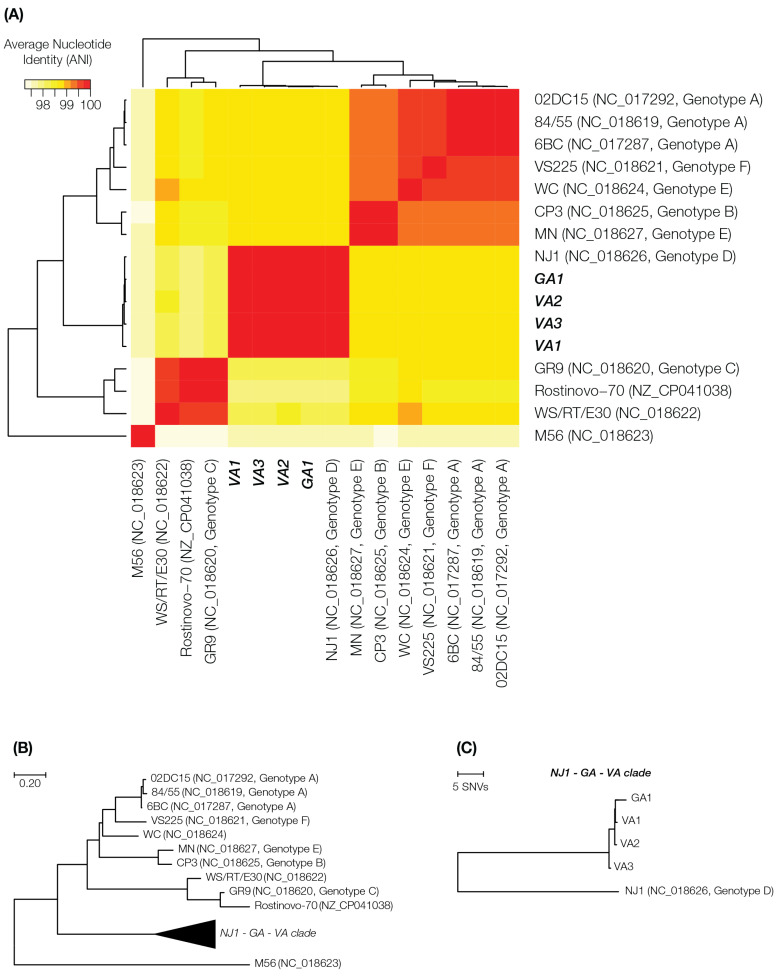
** Comparison of whole genome sequences of outbreak-associated *C. psittaci* isolates and reference genomes.** (A) Whole genome average nucleotide distance phylogenetic tree. (B) Average nucleotide identity heatmap with all publicly available reference genomes of *C. psittaci* and the four outbreak isolates.

**Table 1:** Sequencing metadata for the four outbreak isolates.

Isolate Name	GA1	VA1	VA2	VA3
Accession Number	JARFVI000000000	JARFVH000000000	JARFVG000000000	JARFVF000000000
Location	Georgia	Virginia	Virginia	Virginia
Illumina MiSeq Sequencing	✔	✔	✔	✔
ONT Sequencing	✔	X	X	X
PacBio RSII Sequencing	✔	X	✔	✔
Percent of Genome Covered^a^	100	100	100	100
# Contigs^b^	1	3	1	1
Genome Size^b^	1161591	1163341	1161755	1161426
N50^b^	1161591	775497	1161755	1161426

^a^Percent of reference genome NJ1 covered by Illumina reads ^b^Final assemblies used for whole genome comparison

**Table 2:** Number of SNPs per genome identified by pairwise comparison of outbreak isolates and reference NJ1.

	NJ1	GA1	VA1	VA2	VA3
NJ1		63	62	61	62
GA1	63		1	2	1
VA1	62	1		2	1
VA2	61	2	2		2
VA3	62	1	1	2	
