# Supplementary material for: Genomic analysis of Chlamydia psittaci from a multistate zoonotic outbreak in two chicken processing plants
Source: J Genomics. 2023 Aug 19;11:40–4. doi: 10.7150/jgen.86558 (PMC10475345; doi:10.7150/jgen.86558)
Supplement: Supplementary file 1 — Supplementary figure and table. [file jgenv11p0040s1.pdf]

Supplementary Figure 1: Whole genome sequence alignment of genotype D reference (NJ1) and four outbreak isolates (GA1, VA1, VA2, VA3).

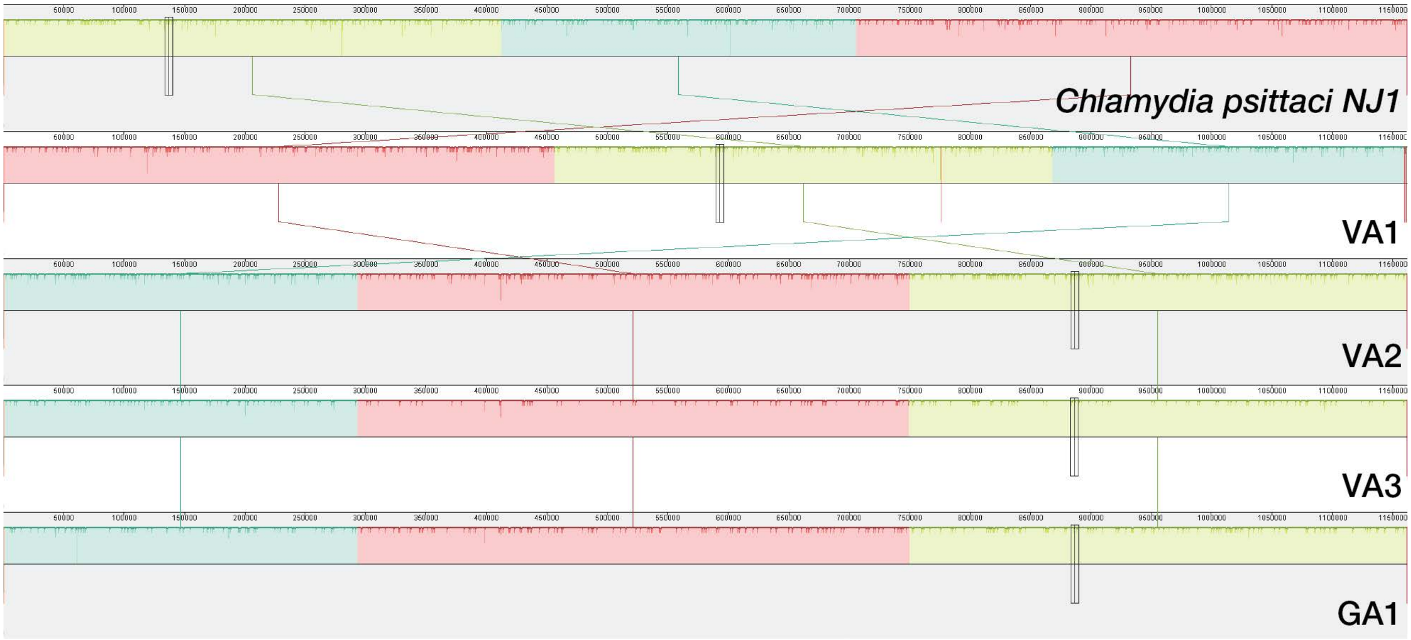

Supplementary Table 1. Detailed information on all single nucleotide polymorphisms (SNPs) identified between the four outbreak isolates and reference sequence NJ1.

Reference Genome	Position	Reference Base	Alternative Base	Variant Quality	Variant Effect	Variant Impact	Gene ID	Gene Symbol	Gene Product	GA1	VA1	VA2	VA3
NC_018626.1	912100	CTT	CT	742	frameshift variant	High	B712_RS03995	.	hypothetical protein	1	1	1	1
NC_018626.1	4273	A	G	742	missense variant	Moderate	B712_RS00020	.	transcript cleavage factor	1	1	1	1
NC_018626.1	53469	C	A	742	missense variant	Moderate	B712_RS00225	.	protein arginine kinase	1	1	1	1
NC_018626.1	82817	A	C	742	missense variant	Moderate	B712_RS00360	.	RsmB/NOP family class I SAM-depende	1	1	1	1
NC_018626.1	86108	T	C	739	missense variant	Moderate	B712_RS00385	.	hypothetical protein	1	1	1	1
NC_018626.1	159906	G	C	742	missense variant	Moderate	B712_RS00785	.	RMD1 family protein	1	1	1	1
NC_018626.1	203571	C	A	739	missense variant	Moderate	B712_RS00990	.	S41 family peptidase	1	1	1	1
NC_018626.1	239421	C	A	739	missense variant	Moderate	B712_RS01165	.	membrane protein	1	1	1	1
NC_018626.1	254992	G	T	742	missense variant	Moderate	B712_RS01250	.	VacB/RNase II family 3'-5' exoribonucle	1	1	1	1
NC_018626.1	267120	A	C	742	missense variant	Moderate	B712_RS01300	.	hypothetical protein	1	1	1	1
NC_018626.1	301930	A	C	742	missense variant	Moderate	B712_RS01415	.	autotransporter domain-containing pro	1	1	1	1
NC_018626.1	313092	A	G	739	missense variant	Moderate	B712_RS01435	.	chemotaxis protein	1	1	1	1
NC_018626.1	353580	T	G	742	missense variant	Moderate	B712_RS01570	.	triose-phosphate isomerase	1	1	1	1
NC_018626.1	417629	G	T	742	missense variant	Moderate	B712_RS01875	.	UDP-N-acetylmuramoyl-L-alanyl-D-glut	1	1	1	1
NC_018626.1	467790	A	G	742	missense variant	Moderate	B712_RS02115	.	4-hydroxy-3-methylbut-2-en-1-yl diphc	1	1	1	1
NC_018626.1	480338	G	T	742	missense variant	Moderate	B712_RS02170	lepA	elongation factor 4	1	1	1	1
NC_018626.1	535108	G	T	742	missense variant	Moderate	B712_RS02400	.	hypothetical protein	1	1	1	1
NC_018626.1	561045	A	T	739	missense variant	Moderate	B712_RS02505	.	hypothetical protein	1	1	1	1
NC_018626.1	580345	G	C	739	missense variant	Moderate	B712_RS02585	.	4-hydroxybenzoate octaprenyltransfera	1	1	1	1
NC_018626.1	636643	C	T	742	missense variant	Moderate	B712_RS02825	.	LifA/Efa1-related large cytotoxin	1	1	1	1
NC_018626.1	639193	A	G	739	missense variant	Moderate	B712_RS02840	.	MAC/perforin family protein	1	1	1	1
NC_018626.1	640838	C	A	742	missense variant	Moderate	B712_RS02840	.	MAC/perforin family protein	1	1	1	1
NC_018626.1	685663	G	A	742	missense variant	Moderate	B712_RS03055	.	FAD-dependent monooxygenase	1	1	1	1
NC_018626.1	715996	A	C	742	missense variant	Moderate	B712_RS03160	.	DUF1389 domain-containing protein	1	1	1	1
NC_018626.1	759378	T	G	739	missense variant	Moderate	B712_RS03335	.	lauroyl acyltransferase	1	1	1	1
NC_018626.1	870418	G	T	742	missense variant	Moderate	B712_RS03830	.	translation initiation factor IF-3	1	1	1	1
NC_018626.1	917044	T	G	739	missense variant	Moderate	B712_RS04010	.	autotransporter domain-containing pro	1	1	1	1
NC_018626.1	935862	C	A	221	missense variant	Moderate	B712_RS04095	pgsA	CDP-diacylglycerol--glycerol-3-phospha	1	0	0	0
NC_018626.1	995968	A	C	742	missense variant	Moderate	B712_RS04360	mfd	transcription-repair coupling factor	1	1	1	1
NC_018626.1	1047391	C	T	742	missense variant	Moderate	B712_RS04580	.	phosphoenolpyruvate carboxykinase ((	1	1	1	1
NC_018626.1	1120005	C	T	739	missense variant	Moderate	B712_RS04870	.	thio:disulfide interchange protein	1	1	1	1
NC_018626.1	619462	G	C	739	stop lost & splice region variant	High	B712_RS02790	.	DUF648 domain-containing protein	1	1	1	1
NC_018637.1	1382	A	T	742	synonymous variant	Low	B712_RS05105	.	.	1	1	1	1
NC_018626.1	3243	T	G	742	synonymous variant	Low	B712_RS00020	.	transcript cleavage factor	1	1	1	1
NC_018626.1	10727	A	G	742	synonymous variant	Low	B712_RS00040	recB	exodeoxyribonuclease V subunit beta	1	1	1	1
NC_018626.1	12348	G	A	739	synonymous variant	Low	B712_RS00045	.	exodeoxyribonuclease V subunit gamm	1	1	1	1
NC_018626.1	17323	C	T	742	synonymous variant	Low	B712_RS00060	rpsD	30S ribosomal protein S4	1	1	1	1
NC_018626.1	69413	A	G	739	synonymous variant	Low	B712_RS00305	.	polysaccharide deacetylase family prot	1	1	1	1
NC_018626.1	256849	G	A	742	synonymous variant	Low	B712_RS01255	dnaK	molecular chaperone DnaK	1	1	1	1
NC_018626.1	302390	A	C	742	synonymous variant	Low	B712_RS01415	.	autotransporter domain-containing pro	1	1	1	1
NC_018626.1	328645	G	T	742	synonymous variant	Low	B712_RS01460	.	autotransporter domain-containing pro	1	1	1	1
NC_018626.1	330136	C	G	739	synonymous variant	Low	B712_RS01475	gatA	Asp-tRNA(Asn)/Glu-tRNA(Gln) amidotr	1	1	1	1
NC_018626.1	409143	G	T	742	synonymous variant	Low	B712_RS01825	.	Na(+)-transporting NADH-quinone redu	1	1	1	1
NC_018626.1	440582	T	C	742	synonymous variant	Low	B712_RS01990	.	hypothetical protein	1	1	1	1
NC_018626.1	517751	G	A	742	synonymous variant	Low	B712_RS02325	infB	translation initiation factor IF-2	1	1	1	1
NC_018626.1	614046	G	C	742	synonymous variant	Low	B712_RS02765	.	IncA family protein	1	1	1	1
NC_018626.1	651963	T	C	742	synonymous variant	Low	B712_RS02890	.	tRNA-guanosine(34) transglycosylase	1	1	1	1
NC_018626.1	683862	G	A	742	synonymous variant	Low	B712_RS03045	.	leucine--tRNA ligase	1	1	1	1
NC_018626.1	1051082	T	G	742	synonymous variant	Low	B712_RS04590	.	DEAD/DEAH box helicase	1	1	1	1
NC_018626.1	1052051	G	T	742	synonymous variant	Low	B712_RS04590	.	DEAD/DEAH box helicase	1	1	1	1
NC_018626.1	1126032	A	G	739	synonymous variant	Low	B712_RS04910	.	peroxiredoxin	1	1	1	1
NC_018626.1	1128456	A	C	739	synonymous variant	Low	B712_RS04925	.	DUF1343 domain-containing protein	1	1	1	1
NC_018626.1	201607	G	C	739	upstream gene variant	Modifier	B712_RS00970	.	hypothetical protein	1	1	1	1
NC_018626.1	340900	G	A	739	upstream gene variant	Modifier	B712_RS01500	.	ribonuclease HIII	1	1	1	1
NC_018626.1	344029	A	C	742	upstream gene variant	Modifier	B712_RS01510	.	hypothetical protein	1	1	1	1
NC_018626.1	531538	T	C	739	upstream gene variant	Modifier	B712_RS02370	.	hypothetical protein	1	1	1	1
NC_018626.1	601583	C	T	17.8704	upstream gene variant	Modifier	B712_RS02710	pgl	6-phosphogluconolactonase	1	1	1	0

NC_018626.1	606083	C	A	742	upstream gene variant	Modifier	B712_RS02715	.	glucose-6-phosphate dehydrogenase	1	1	1	1
NC_018626.1	612267	TT	TTAT	140	upstream gene variant	Modifier	B712_RS02745	.	hypothetical protein	1	1	1	1
NC_018626.1	740285	G	A	742	upstream gene variant	Modifier	B712_RS03240	rplS	50S ribosomal protein L19	1	1	1	1
NC_018626.1	973464	C	T	739	upstream gene variant	Modifier	B712_RS04245	.	hypothetical protein	1	1	1	1
NC_018626.1	1013557	C	A	742	upstream gene variant	Modifier	B712_RS04430	.	DNA translocase FtsK	1	1	1	1
NC_018626.1	1072795	G	T	742	upstream gene variant	Modifier	B712_RS04665	.	phage holin family protein	1	1	1	1
NC_018626.1	277628	.	I743068-746765		inframe insertion	Moderate	B712_RS01350	.	membrane protein	1	1	1	1
